# Association Study among Comethylation Modules, Genetic Polymorphisms and Clinical Features in Mexican Teenagers with Eating Disorders: Preliminary Results

**DOI:** 10.3390/nu13093210

**Published:** 2021-09-15

**Authors:** Germán Alberto Nolasco-Rosales, José Jaime Martínez-Magaña, Isela Esther Juárez-Rojop, Thelma Beatriz González-Castro, Carlos Alfonso Tovilla-Zarate, Ana Rosa García, Emmanuel Sarmiento, David Ruiz-Ramos, Alma Delia Genis-Mendoza, Humberto Nicolini

**Affiliations:** 1 Biomedical Postgraduate Program, Academic Division of Health Sciences, Juárez Autonomous University of Tabasco, Villahermosa 86000, Mexico; ganr_1277@live.com.mx (G.A.N.-R.); iselajuarezrojop@hotmail.com (I.E.J.-R.); daruiz_914@hotmail.com (D.R.-R.); 2 Genomics of Psychiatric and Neurodegenerative Diseases Laboratory, National Institute of Genomic Medicine (INMEGEN), Mexico City 01090, Mexico; martinezmaganajjaime@gmail.com; 3 Genomics Laboratory, Academic Division Jalpa de Mendez, Juárez Autonomous University of Tabasco, Jalpa de Mendez 86200, Mexico; thelma.glez.castro@gmail.com; 4 Genomics Laboratory, Comalcalco Multidisciplinary Academic Division, Juárez Autonomous University of Tabasco, Villahermosa 86000, Mexico; alfonso_tovillaz@yahoo.com.mx; 5 Children’s Psychiatric Hospital “Dr. Juan N. Navarro”, Mexico City 01090, Mexico; anarosagarciab@gmail.com (A.R.G.); emmanuelsarmientoh@hotmail.com (E.S.)

**Keywords:** comethylation modules, genetic polymorphisms, eating disorders, WGCNA

## Abstract

Eating disorders are psychiatric disorders characterized by disturbed eating behaviors. They have a complex etiology in which genetic and environmental factors interact. Analyzing gene-environment interactions could help us to identify the mechanisms involved in the etiology of such conditions. For example, comethylation module analysis could detect the small effects of epigenetic interactions, reflecting the influence of environmental factors. We used MethylationEPIC and Psycharray microarrays to determine DNA methylation levels and genotype from 63 teenagers with eating disorders. We identified 11 comethylation modules in WGCNA (Weighted Gene Correlation Network Analysis) and correlated them with single nucleotide polymorphisms (SNP) and clinical features in our subjects. Two comethylation modules correlated with clinical features (BMI and height) in our sample and with SNPs associated with these phenotypes. One of these comethylation modules (yellow) correlated with BMI and rs10494217 polymorphism (associated with waist-hip ratio). Another module (black) was correlated with height, rs9349206, rs11761528, and rs17726787 SNPs; these polymorphisms were associated with height in previous GWAS. Our data suggest that genetic variations could alter epigenetics, and that these perturbations could be reflected as variations in clinical features.

## 1. Introduction

Eating disorders (EDs) are severe psychiatric disorders characterized by disturbances of eating behavior, affecting the health and quality of life of individuals. These disorders have an early teenage onset and a hereditary component [1]. EDs have a complex etiology in which genetic and environmental factors interact [2]. Genome-wide association studies (GWAS) and other genetic studies have revealed loci and single nucleotide polymorphisms (SNP) associated with ED [1,3,4]. The clinical characteristics of EDs have been associated in genetic studies. In this sense, significant genetic correlations have been reported in anorexia nervosa with psychiatric disorders, physical activity and metabolic, lipid and anthropometric traits [5]. In addition, genetic associations between ED and substance use have been described [6]. Additionally, the genetic-environment relationship in ED has been studied through DNA methylation, reporting perturbations in the methylation levels of some genes (*DRD2*, *SLC6A3*, *POMC*, *OXTR*, among others) [2,7,8]. However, genes do not function alone: on average, each gene is estimated to interact with another four or eight genes, and to be involved with 10 biological functions. Furthermore, recent studies suggest that gene networks provide the potential to identify hundreds of disease-related genes [9]. Analyzing gene-environment interactions in EDs could help us to identify the mechanisms involved in their etiology. Nowadays, new technologies evaluating thousands of genes apply statistic approaches that integrate different information sources from gene interactions (e.g., comethylation module construction) [10]. Comethylation modules are clusters of highly interconnected CpG sites. These modules are detected through the construction of a correlation network. Correlation networks are used to analyze large, high-dimensional data sets. These correlation networks are constructed on the basis of correlations among quantitative measurements (e.g., gene expression profiles, methylation levels) [11]. Comethylation modules are formed by using methylation data as quantitative measurements of gene-environment interactions [10]. Additionally, comethylation modules alleviate various testing problems which are inherent to microarray data analyses, and have been found to be useful for describing pairwise relationships among methylated genes [9,10,11]. In brief, comethylation modules (1) consider all genes as interconnected, (2) identify groups of CpG sites with similar methylation levels, (3) increase statistical power, and (4) detect small effects of epigenetic interactions [9,10,11]. Thus, evaluating correlations among genetic factors, comethylation modules, and clinical features in EDs could be a means by which to identify biological markers in such disorders. The objective of the present study was to detect comethylation modules from DNA methylation samples from children and teenagers with an ED, and to correlate these modules with clinical features and genetic variability.

## 2. Materials and Methods

### 2.1. Study Population

We included 63 subjects diagnosed with anorexia nervosa (AN), bulimia nervosa, (BN) or binge eating disorder (BED) using DSM 5 criteria [12]. Individuals were recruited in the outpatient center of the Children’s Psychiatric Hospital “Dr. Juan N. Navarro” from May 2014 to August 2016. Inclusion criteria were subjects with at least three generations of Mexican lineage, 12–18 years of age, and individuals not using psychotropic or psychoactive drugs. The clinical features of the sample are descripted in Table 1.

This study followed the principles of the Declaration of Helsinki. Sample recollection and processing were approved by the Ethics Committee of the Children’s Psychiatric Hospital “Dr. Juan N. Navarro” with approval No. II3/01/0913 (11 October 2017), and by the Ethics Committee of the National Institute of Genomic Medicine (INMEGEN) with approval No. 06/2018/I.

### 2.2. Evaluation Instruments

BED was screened with the QEWP-R (Questionnaire on Eating and Weight Pattern-Revised) [13]. AN was screened with EAT-26 (Eating Attitudes Test) [14]. We evaluated the presence of psychiatric comorbidity with the Spanish version of MINI Kid (Mini International Neuropsychiatric Interview for Children and Adolescent) [15]. A pedopsychiatrist performed all ED diagnoses.

### 2.3. DNA Extraction and Microarray Analysis

After diagnostic evaluation of each individual, a blood sample was collected using an EDTA tube; DNA was subsequently extracted from this sample. We used the salting-out method from the Gentra Puregene Blood (Qiagen, Germantown, MD, USA) commercial kit. DNA extraction quality and integrity were evaluated by analysis with a NanoDrop spectrophotometer (Thermofisher, Waltham, MA, USA) and 2% agarose gel. DNA samples met the following quality criteria: visible genomic DNA band, 230/260 and 260/230 ratios >1.8, concentration >50 ng/µL, and no signs of DNA degradation. For genotypification, we hybridized DNA with commercial microarray Infinium Psycharray Beadchip (Illumina, San Diego, CA, USA). For methylation analysis, DNA was bisulfite converted using an EZ DNA Methylation Kit (Zymo Research, Irvine, CA, USA). Converted DNA was hybridized with the Infinium MethylationEPIC BeadChip (Illumina, San Diego, CA, USA). Each microarray was processed in the Microarray and Expression Unit of the National Institute of Genomic Medicine.

### 2.4. Quality Control of Genotypification Data

We transformed fluorescence intensities from the Psycharray into genotypes using the GenomeStudio (v. 2.0) software, and quality control was done with the PLINK (v. 1.9) toolset [16]. We eliminated: (1) SNPs with less than 95% genotype calls, (2) individuals with less than 95% genotype calls, (3) individuals with sex discrepancy, (4) SNPs located in chromosomes X and Y, (5) SNPs with less than 0.05 minor allele frequency (MAF), (6) SNPs deviating from Hardy-Weinberger equilibrium (*p* < 1 × 10^−6^), and (7) SNPs with A/T and C/G alleles. Subsequently, filtrated data were exported to the R (v. 4.0) software [17], and we removed SNPs with missing data and SNPs without homozygous individuals to minor alleles. Only 193,314 SNPs passed the quality control.

### 2.5. Quality Control of DNA Methylation Data

The fluorescence intensities of the MethylationEPIC microarray were transformed into *idat* files, which were filtered with ChAMP pipeline (v.2.18) [18] for R (v. 4.0) software. Quality control removed: (1) probes with detection *p*-value > 0.01, (2) probes with <3 beads in at least 5% of samples per probe, (3) non-CpG probes, (4) multihit probes, (5) probes located in chromosome X and Y, and (6) individuals with sex discrepancy in their genotypification data. We converted filtered methylation data into β-values, which were normalized using the BMIQ (Beta-Mixture Quantile Normalization) method [19]. Afterwards, we evaluated the presence of the batch effect with a singular value decomposition (SVD) method. We preserved CpG sites with standard deviation (SD) > 0.05, keeping 105,393 sites. Likewise, we made many cut points in SD (0.05, 0.06, 0.07, 0.08, 0.09, 0.10, 0.15, 0.20) to find an optimal point for the construction of comethylation modules.

### 2.6. Comethylation Modules Construction

In order to identify comethylation modules, we processed methylation values with the WGCNA (Weighted correlation network analysis) package [11,20] (R software). Later, we applied the means method to achieve hierarchical clustering, and eliminated individuals with atypical samples. For the final analysis, we only considered 50 subjects. Furthermore, an analysis of network topology was used to determine a soft-thresholding power less than 20 with suitable independence (>0.8) and mean connectivity (<1000). The CpG sites with SD > 0.06 in their methylation values had the best network topology. We applied *blockwiseModules* function (WGCNA package [11,20]) to detect comethylation modules, using a minimum module size of 175 and a threshold of 20. In a subsequent analysis, we discarded the CpG sites clustered in the grey module, and identified a new set of modules. The 11 constructed comethylation modules included 11,418 CpG sites. Each module was automatically assigned a color by WGCNA, indicating its size. The grey module was discarded from further analysis as it groups unassigned CpG sites to other modules; thus, these sites are unrelated.

### 2.7. Enrichment Analysis of Modules

The CpG sites inside modules were annotated using IlluminaHumanMethylationEPICanno.ilm10b4.hg19 package [21]. Genes of CpG sites were extracted and enriched using the WebGestalt online tool [22]. We accomplished the enrichments with Over-Representation Analysis of KEGG Database (Kyoto Encyclopedia of Genes and Genomes) [23]; this was considered to be significant with an adjusted *p*-value by FDR ≤ 0.05.

### 2.8. Correlation of Comethylation Modules with Clinical Features and SNPs

The eigengene of each comethylation module was correlated with clinical data and SNPs using Pearson’s correlations. We calculated the R^2^ and *p* values with *cor* and *corPvalueStudent* functions and set the significant value *p* < 5 × 10^−3^ for clinical data and *p* < 5 × 10^−8^ for SNPs. In order to find associations between SNPs and phenotypes, we used the PheWAS tool on the GWAS Atlas website [24]. Associations were considered to be significant for any phenotype with a *p* < 1 × 10^−10^.

## 3. Results

### 3.1. Description of Comethylation Modules

There were 11 modules in our study. Modules were turquoise (5073 sties), blue (2928 sites), brown (193 sites), yellow (166 sites), green (151 sites), red (150 sites), black (148 sites), pink (145 sites), magenta (135 sites), purple (111 sites), and grey (2218 sites). The CpG sites of these modules were located in 4005 genes. According to relative position to gene, the gene body was the most common annotated location, ranging from 40 sites (56.34%) in the purple module to 2430 sites (69.27%) in the turquoise module. Table 2 shows the details of functional annotation of CpG sites from comethylation modules regarding gene location.

Concerning the distribution of CpG sites with respect to CpG islands, a majority of comethylation modules corresponded to Open Sea (71.11–84.56%, 85–4207 sites); on the other hand, the purple comethylation module had a high percentage of CpG sites annotated on islands (15.32%, 17 sites) (Table 3). CpG sites in comethylation modules were heterogeneously distributed among chromosomes (Table S1). Additionally, we observed two groups of comethylation modules given the methylation levels from beta values. One group had partially methylated values (0.2 < β value < 0.8) (turquoise, blue and purple), while the other group had hypermethylated values (β value ≥ 0.8) (brown, yellow, green, red, black, pink and magenta) (Table S2).

### 3.2. Enriched Pathways on Each Module

We found significant enriched pathways of genes annotated on the CpG sites from turquoise and blue modules (Table S3). Genes in the turquoise module were enriched for the longevity regulating pathway (adjusted *p* value = 0.0047), GnRH (Gonadotropin-releasing hormone) signaling pathway (adjusted *p* value = 0.0042), glioma (adjusted *p* value = 0.0126), cholinergic synapse (adjusted *p* value = 0.0091), human cytomegalovirus infection (adjusted *p* value = 0.0126), and endocytosis (adjusted *p* value = 0.0126). Genes from blue comethylation module were enriched in pathways for Th1 and Th2 cell differentiation (adjusted *p* value = 6.8672 × 10^−7^), allograft rejection (adjusted *p* value = 0.0185), endometrial cancer (adjusted *p* value = 0.0111), and TNF signaling pathway (adjusted *p* value = 0.0007). Another enrichment pathways within the same module included the AGE-RAGE signaling pathway in diabetic complications (adjusted *p* value = 0.0033), phosphatidylinositol signaling system (adjusted *p* value = 0.0074), glioma (adjusted *p* value = 0.0365), longevity regulating pathway (adjusted *p* value = 0.0325), human cytomegalovirus infection (adjusted *p* value = 0.0008), and focal adhesion (adjusted *p* value = 0.0039).

### 3.3. Correlations of Modules with Clinical Features in Our Population

**Figure 1:**
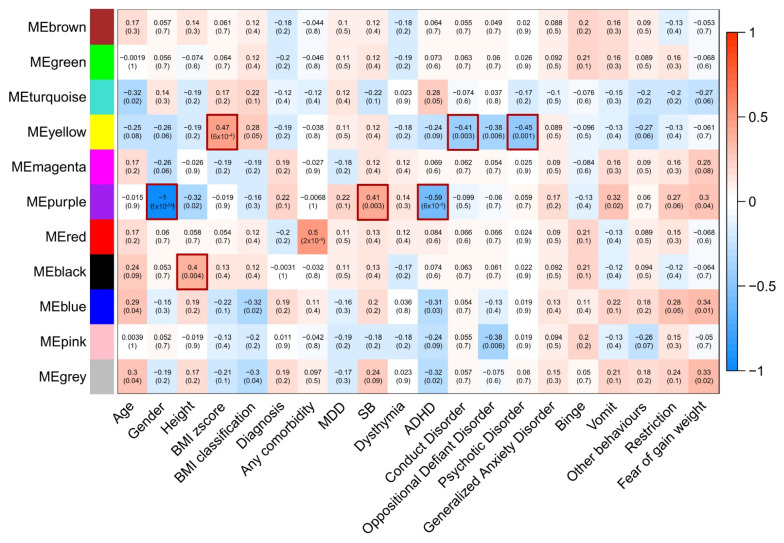
Correlations between modules and clinical features. MDD: major depressive disorder. SB: suicidal behavior. ADHD: attention deficit hyperactivity disorder. Red borders indicate significant correlations. Significance indicates *p*-values <5 × 10^−3^.

In our results, seven clinical features and comorbidities correlated with different comethylation modules (Figure 1). The yellow comethylation module correlated with body mass index (BMI) zscore (R^2^ = 0.47, *p* = 0.0006), conduct disorder (R^2^ = −0.41, *p* = 0.0030), and psychotic disorder (R^2^ = −0.45, *p* = 0.0010). Meanwhile, the purple comethylation module correlated with gender (R^2^ = −1, *p* < 1 × 10^−50^), suicidal behavior (R^2^ = 0.41, *p* = 0.0030), and attention-deficit/hyperactivity disorder (R^2^ = −0.59, *p* = 6 ×10^−6^). Finally, the black module correlated with height (R^2^ = 0.4, *p* = 0.0040). Notably, clinical features did not correlate with more than one module at a time.

### 3.4. Correlations of SNPs with Modules

Seven comethylation modules had correlations with any SNP (brown, green, yellow, magenta, red, black and pink) (Table S4). SNPs were located mostly in intronic regions, ranging from 33.96% in the red module (18 SNPs) to 55.56% in the yellow module (15 SNPs). Another frequent location was intergenic regions, ranging from 14.81% in the yellow module (4 SNPs) to 31.71% in the black module (13 SNPs). The most frequent location in the green module was intergenic regions (28.13%, 9 SNPs), followed by intronic regions (21.88%, 7 SNPs).

The most correlated SNPs (89.95%, 206 SNPs) were in nonprotein-coding transcript regions, while 10.05% (23 SNPs) were in protein-coding regions (synonymous and missense). Seventeen SNPs (7.42%) were annotated as missense variants; the red and black modules had four missense SNPs each. Meanwhile, six correlated SNPs (2.62%) were annotated as synonymous variants, with two SNPs per module (brown, yellow, and red). We observed 10 correlated SNPs (4.37%) in regulatory regions, although no SNPs were found in the yellow comethylation module. The magenta comethylation module had no SNPs annotated in the upstream and downstream regions. Finally, correlated SNPs annotated in 3′ untranslated regions (UTR) were the least frequent (2 SNPs, 0.87%), found within the magenta and red modules (Table 4).

### 3.5. Correlated SNP PheWAS

**Figure 2:**
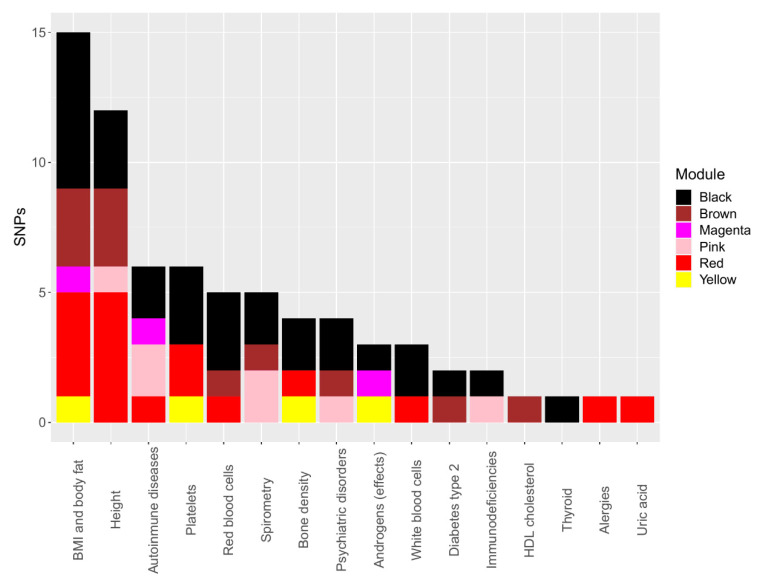
Phenotypes associated with correlated single nucleotide polymorphisms (SNP) in PheWAS.

Regarding clinical features, BMI, body fat, and height were the most frequent phenotypes associated with SNPs (Figure 2).

Concerning psychiatric disorders, we found several SNPs to be associated with three comethylation modules. The brown comethylation module had a SNP associated with depressive symptoms and neuroticism (rs4598994). Meanwhile, the pink comethylation module was associated with depressive affect (rs4800995); two SNPs were associated with schizophrenia (rs3129012 and rs356971) in the black comethylation module. Moreover, seven correlated SNPs with four comethylation modules were associated with autoimmune diseases. The magenta comethylation module was associated with rheumatoid arthritis and Crohn’s disease (rs1893217). Likewise, the red (rs3095345) and pink (rs9267546 and rs9267547) comethylation modules were associated with rheumatoid arthritis and type 1 diabetes. The black module was associated with primary sclerosing cholangitis (rs3129012 and rs356971), autoimmune vitiligo, and systemic lupus erythematosus (rs356971). Finally, the yellow and black comethylation modules were correlated with clinical features in our population (BMI zscore, conduct disorder, psychotic disorder, and height); likewise, these comethylation modules were correlated with SNPs associated with similar phenotypes. Meanwhile, the yellow module correlated with one SNP (rs10494217) associated with the waist–hip ratio in PheWAS, and the black comethylation module correlated with three SNPs associated with height (rs9349206, rs11761528 and rs17726787).

## 4. Discussion

Several studies have evaluated the clinical features, genetic variants, and single DNA methylation sites involved in ED, but none has considered these factors together to date [1,5,7]. As such, little information is available about the integration of these different levels of biological information. As far as we know, this is the first study to integrate clinical features, genetic variants, and DNA methylation using a comethylation network analysis in teenagers with EDs.

BMI is an important clinical characteristic of the individuals diagnosed with ED, and it has a high impact on metabolic profiles [25]. Low BMI is a diagnosis criterion for anorexia nervosa [12]; on the other hand, bulimia nervosa and binge-eating disorder are related to risk of overweight/obesity [26]. In our analysis, the yellow module could be important in the changes in BMI found in individuals with EDs. The yellow module was correlated with BMI and rs10494217. This SNP is a missense genetic variant changing a histidine aminoacid for an asparagine in position 50 of *TBX15* gene (p.His50Asn). Previously, GWAS associated rs10494217 with waist-hip index, a variable related to BMI [27]. The *TBX15* gene is a member of the T-box gene family, i.e., transcriptional regulators which play an important role in the development of skeletal elements of limbs, vertebral column, and head, as well as other organs [28,29]. Likewise, this gene was reported as a regulator of metabolism of adipose tissue and muscle fibers, and shown to indirectly regulate body fat and BMI [30,31,32]. *TBX15* is highly expressed in adipose tissue, and it binds to the promoter of *PRDM16* gene. *PRDM16* is essential for the browning of adipose tissue; reduced expression of its protein promotes obesity with high-fat diet and increases visceral fat [33]. As a missense variant, rs10494217 could reduce the binding of *TBX15* protein to the promoter of *PRDM16*, and thus disturb adipose tissue function and alter BMI in individuals diagnosed with an ED. A possible alteration of *PRDM16* expression could induce epigenetic reprogramming, as it is found in the yellow module in this study. CpG sites of the yellow module were enriched in alpha-linolenic acid metabolism (*PLA2G4E* and *PLB1*) and VEGF signaling pathway (*AKT3*, *NFATC2*, *PLA2G4E* and *SHC2*); both pathways are involved in adipose tissue function and BMI [34,35,36,37].

The black module could also be related to BMI in ED-diagnosed individuals. This module was correlated with rs11761528, a SNP associated with BMI and androsterone sulfate metabolism [27,38]. rs11761528 is an intronic polymorphism of the *ZKSCAN5* gene. There is little information about *ZKSCAN5* (zinc finger with KRAB and SCAN domains 5) and its mechanism; however, animal models suggest that this gene is correlated with adipocyte volume, systolic blood pressure, and cardiac mass [39]. Similarly, rs17726787 was previously associated with height and trunk fat-free mass in GWAS [24,40]. This SNP is an intronic variant of the *CELF1* gene. Disturbances in gene expression of *CELF1* are related with cardiopathies [41,42,43]. The black module was enriched in mTOR signaling pathway (*IGF1R*, *LPIN1* and *RPS6KA2*), suggesting that genetic variations like rs11761528 and rs17726787 could alter the epigenetics of this pathway. The mTOR signaling pathway is essential for cardiac development [44,45]. Heart complications are frequent in anorexia nervosa patients, reaching 80% in some studies. Severe anorexia nervosa can change cardiac structure, although most structural abnormalities are reversible [46,47]. Nevertheless, there is a lack of analyses which explore the relationships between altered genes in the module, genetic variation, and cardiopathies in ED-diagnosed individuals.

Although schizophrenia was not correlated in our sample, we found genes and SNPs associated with the disorder. Schizophrenia and other psychiatric disorders are associated with anorexia nervosa [3,5]. Also, there is reportedly a high prevalence of schizophrenia among individuals with eating disorders [48]. CpG sites conforming the black module were enriched in morphine addiction (*GABBR2*, *GABRP* and *PDE4B*), and polymorphisms of the *PDE4B* gene have been associated with susceptibility to schizophrenia [49]. rs356971 and rs3129012 SNPs were correlated with the black module; these SNPs are associated with schizophrenia [50] and waist–hip index [27]. Furthermore, rs356971 and rs3129012 are associated with phenotypes related with the immunological system, hemoglobin concentration, white blood cells, and platelet count [51]. These SNPs are also associated with primary sclerosing cholangitis [52], autoimmune vitiligo [53], IgA deficiency [54], and systemic lupus erythematosus [55]. Immune-mediated mechanisms have been suggested in the development of EDs; an increased risk for autoimmune diseases in EDs has been reported [56,57]. Likewise, a locus in chromosome 12 was associated with anorexia nervosa, diabetes type 1, and autoimmune diseases [3].

Some modules could have comethylation of CpG sites which was not altered by a genetic effect in individuals diagnosed with an ED, like the turquoise and blue modules. These modules were enriched in pathways associated with the immunological system (Th1 and Th2 cell differentiation, TNF signaling pathway, focal adhesion). The same modules were also enriched in pathways related with development status. The construction of these modules could be influenced by the developmental stage of individuals in the sample, i.e., mainly teenagers [58]. One pathway is the GnRH (Gonadotropin Release Hormone) signaling pathway, which is activated at the beginning of pubertal development, and it depends on neuroendocrine signaling [59,60,61]. Another enriched pathway was associated with adiponectin (adiponectin/CaMKK/AMPK) [62,63]. Many authors suggest that adiponectin levels change with pubertal development [64,65]. Also, partial methylation of these modules suggests transcriptional activation of these pathways. The detection of these modules is more likely to be an effect of background epigenetic alterations and the cell development stage in the tissue (white blood cells) used for the analysis.

Our study has some limitations. Firstly, we note the absence of a control group. However, in this exploratory study, our primary aim was to detect comethylation modules in ED patients and assess the relationship between such modules and clinical phenotypes in ED. Second, we had a small sample with many variables evaluated. Although these conditions could affect the statistic power of our analysis, comethylation modules aggregate covarying CpGs and evaluate grouped CpGs, reducing the number of tests needed. Besides, WGCNA requires at least 20 samples to construct biologically meaningful modules [66]. Third, our data was from a sample made up of Mexican teenagers; therefore, our results should not be applied to all populations with EDs.

## 5. Conclusions

This is the first study integrating clinical features, genetic variants, and DNA methylation using comethylation network analysis in teenagers with ED. Our findings showed that two comethylation modules correlated with physical features as well as with SNPs previously associated with metabolic and psychiatric phenotypes. These data suggest that genetic variations could alter epigenetics, and that these perturbations could be reflected as variations in clinical features.

## Figures and Tables

**Table 1:** Clinical features of study population.

Features	Sample (*n* = 50)
Age (years)	13.98 ± 1.74
Gender	
Male	13 (26)
Female	37 (74)
Body Mass Index (BMI) zscore	1.03 ± 0.97
BMI classification	
Underweight	1 (2)
Normal weight	20 (40)
Overweight	11 (22)
Obesity	18 (36)
Diagnosis	
Binge eating disorder	17 (34)
Bulimia nervosa	22 (44)
Anorexia nervosa	11 (22)
Comorbidities	
Any	46 (92)
Major depressive disorder	21 (42)
Suicide behavior	16 (32)
Dysthymia disorder	18 (36)
Attention-Deficit/Hyperactivity Disorder	15 (30)
Generalized Anxiety Disorder	10 (20)
Oppositional Defiant Disorder	6 (12)
Conduct Disorder	5 (10)
Psychotic Disorder	5 (10)
Eating Attitudes	
Fear of gain weight	35 (70)
Binge	34 (68)
Restriction	24 (48)
Vomit	21 (42)
Other behaviors	10 (20)

Features of subjects who satisfied inclusion criteria. Continuous variables are expressed as mean ± standard deviation. Categorical variables are expressed as *n* (%).

**Table 2:** Comethylation module CpG sites classification.

Module		TSS1500	TSS200	5′UTR	Body	1stExon	ExonBnd	3′UTR
Turquoise		359 (10.23)	97 (2.77)	436 (12.43)	2430 (69.27)	33 (0.94)	37 (1.05)	116 (3.31)
Blue		158 (7.85)	75 (3.72)	309 (15.34)	1366 (67.83)	23 (1.14)	16 (0.79)	67 (3.33)
Brown		14 (10.77)	4 (3.08)	23 (17.69)	78 (60.00)	2 (1.54)	3 (2.31)	6 (4.62)
Yellow		13 (12.15)	6 (5.61)	13 (12.15)	69 (64.49)	2 (1.87)	0 (0)	4 (3.74)
Green		11 (11.22)	3 (3.06)	13 (13.27)	68 (69.39)	1 (1.02)	1 (1.02)	1 (1.02)
Red		20 (18.52)	1 (0.93)	15 (13.89)	66 (61.11)	2 (1.85)	1 (0.93)	3 (2.78)
Black		11 (11.22)	2 (2.04)	9 (9.18)	66 (67.35)	2 (2.04)	2 (2.04)	6 (6.12)
Pink		13 (13.00)	2 (2.00)	9 (9.00)	71 (71.00)	2 (2.00)	1 (1.00)	2 (2.00)
Magenta		10 (11.36)	4 (4.55)	12 (13.64)	56 (63.64)	1 (1.14)	0 (0)	5 (5.68)
Purple		8 (11.27)	5 (7.04)	8 (11.27)	40 (56.34)	1 (1.41)	2 (2.82)	7 (9.86)

CpG sites were annotated with the IlluminaHumanMethylationEPICanno.ilm10b4.hg19 [21] package. Data expressed as *n* of sites, (%) by rows. TSS: Transcription Start Site. UTR: Untranslated Region. ExonBnd: Exon Boundaries.

**Table 3:** CpG site position with respect to CpG islands.

Module		OpenSea	Island	N Shore	S Shore	N Shelf	S Shelf
Turquoise		4207 (82.93)	13 (0.26)	247 (4.87)	212 (4.18)	202 (3.98)	192 (3.78)
Blue		2476 (84.56)	15 (0.51)	128 (4.37)	83 (2.83)	104 (3.55)	122 (4.17)
Brown		153 (79.27)	6 (3.11)	9 (4.66)	17 (8.81)	4 (2.07)	4 (2.07)
Yellow		125 (75.30)	1 (0.60)	14 (8.43)	10 (6.02)	12 (7.23)	4 (2.41)
Green		114 (75.50)	5 (3.31)	10 (6.62)	8 (5.30)	9 (5.96)	5 (3.31)
Red		121 (80.67)	1 (0.67)	6 (4.00)	2 (1.33)	8 (5.33)	12 (8.00)
Black		115 (77.70)	1 (0.68)	7 (4.73)	6 (4.05)	11 (7.43)	8 (5.41)
Pink		114 (78.62)	6 (4.14)	7 (4.83)	7 (4.83)	8 (5.52)	3 (2.07)
Magenta		96 (71.11)	6 (4.44)	9 (6.67)	9 (6.67)	8 (5.93)	7 (5.19)
Purple		85 (76.58)	17 (15.32)	4 (3.60)	3 (2.70)	1 (0.90)	1 (0.90)
Total		7606 (82.67)	71 (0.77)	441 (4.79)	357 (3.88)	367 (3.99)	358 (3.89)

CpG sites were annotated with the IlluminaHumanMethylationEPICanno.ilm10b4.hg19 [21] package. Data expressed in *n* of sites, (%) by rows.

**Table 4:** Annotations of SNPs correlated with modules.

	Brown	Green	Yellow	Magenta	Red	Black	Pink	Total
								
3′UTR	0 (0.00)	0 (0.00)	0 (0.00)	1 (5.88)	1 (1.89)	0 (0.00)	0 (0.00)	2 (0.89)
Downstream	0 (0.00)	2 (6.25)	1 (3.70)	0 (0.00)	4 (7.55)	1 (2.44)	1 (5.00)	9 (4)
Intergenic	7 (17.95)	9 (28.13)	4 (14.81)	5 (29.41)	13 (24.53)	13 (31.71)	4 (20.00)	55 (24.4)
Intron	19 (48.72)	7 (21.88)	15 (55.56)	7 (41.18)	18 (33.96)	14 (34.15)	7 (35.00)	87 (38.67)
Missense	3 (7.69)	1 (3.13)	2 (7.41)	1 (5.88)	4 (7.55)	4 (9.76)	2 (10.00)	17 (7.56)
Non coding transcript	3 (7.69)	8 (28.13)	2 (7.41)	1 (5.88)	6 (13.21)	5 (14.64)	3 (20.00)	28 (12.44)
Regulatory	1 (2.56)	3 (9.38)	0 (0.00)	2 (11.76)	2 (3.77)	1 (2.44)	1 (5.00)	10 (4.44)
Synonymous	2 (5.13)	0 (0.00)	2 (7.41)	0 (0.00)	2 (3.77)	0 (0.00)	0 (0.00)	6 (2.67)
Upstream	4 (10.26)	1 (3.13)	1 (3.70)	0 (0.00)	2 (3.77)	2 (4.88)	1 (5.00)	11 (4.89)

dbSNP codes were annotated with InfiniumPsychArray-24v1-3_A1_b150_rsids file. Coding regions were annotated with Ensembl Variant Effect Predictor. Data expressed as *n* of SNPs, (%) by columns.

## Data Availability

The data presented in this study are available on request from the corresponding author, which were omitted due to privacy and ethical issues.

## Supplementary Materials

The following are available online at https://www.mdpi.com/article/10.3390/nu13093210/s1, Table S1: CpG sites per chromosome and normalized value per chromosomic size, Table S2: Descriptive statistics of comethylation modules’ beta values, Table S3: Functional enrichments of comethylation modules, Table S4: Characteristics of SNPs correlated with comethylation modules.
